# ESPO AND ACADEMY FOR GERONTOLOGY IN HIGHER EDUCATION SECTION SYMPOSIUM: TEACHING STRATEGIES FOR INTEGRATING DIVERSITY IN AGING EDUCATION PRACTICES

**DOI:** 10.1093/geroni/igac059.034

**Published:** 2022-12-20

**Authors:** YanJhu Su, Shan Qu, Joann Montepare

## Abstract

This symposium intends to echo one of the core values for the GSA 2022 Annual Scientific Meeting — embracing diversity. In an increasingly humanistic society, addressing and accommodating diversity is a constant topic in the field of education. Diversity includes many different factors: race, ethnicity, gender, sexual orientation, socioeconomic status, ability, age, religious or political beliefs. There is no longer just one type of student in the classroom. Therefore, it is very important to adjust our teaching methods to accommodate the diverse backgrounds of students and to help students understand their different fellow classmates. In accordance with the AGHE gerontological education competencies, these authors will provide insightful teaching strategies for integrating diversity in aging education practices. The first speaker will introduce a new pedagogy in promoting learning in diverse classrooms — Sharestart. The second speaker will focus on building community-based partnerships to promote diverse, intergenerational service-learning opportunities for interprofessional gerontology students. The third speaker will discuss their experience on fostering connections across generations in the LGBTQ+ community through intergenerational conversations. The fourth speaker will present humanist values on understanding aging diversity. Finally, the fifth speaker will discuss curriculum mapping as a strategy to teach diversity in gerontology programs.

